# Ten Simple Rules for Editing Wikipedia

**DOI:** 10.1371/journal.pcbi.1000941

**Published:** 2010-09-30

**Authors:** Darren W. Logan, Massimo Sandal, Paul P. Gardner, Magnus Manske, Alex Bateman

**Affiliations:** 1 Wellcome Trust Sanger Institute, Cambridge, United Kingdom; 2 Department of Chemistry, University of Cambridge, Cambridge, United Kingdom

Wikipedia is the world's most successful online encyclopedia, now containing over 3.3 million English language articles. It is probably the largest collection of knowledge ever assembled, and is certainly the most widely accessible. Wikipedia can be edited by anyone with Internet access that chooses to, but does it provide reliable information? A 2005 study by *Nature* found that a selection of Wikipedia articles on scientific subjects were comparable to a professionally edited encyclopedia [1], suggesting a community of volunteers can generate and sustain surprisingly accurate content.

For better or worse, people are guided to Wikipedia when searching the Web for biomedical information [2]. So there is an increasing need for the scientific community to engage with Wikipedia to ensure that the information it contains is accurate and current. For scientists, contributing to Wikipedia is an excellent way of fulfilling public engagement responsibilities and sharing expertise. For example, some Wikipedian scientists have successfully integrated biological data with Wikipedia to promote community annotation [3], [4]. This, in turn, encourages wider access to the linked data via Wikipedia. Others have used the wiki model to develop their own specialist, collaborative databases [5]–[8]. Taking your first steps into Wikipedia can be daunting, but here we provide some tips that should make the editing process go smoothly.

## Rule 1: Register an Account

Although any visitor can edit Wikipedia, creating a user account offers a number of benefits. Firstly, it offers you privacy and security. Though counterintuitive, editors registered under a pseudonymous username actually have greater anonymity than those who edit “anonymously”. A few of us have chosen to associate our accounts with our real identities. Should you choose to forgo pseudonymity on Wikipedia, your entire editing history will be open to indefinite scrutiny by curious Web searchers, including future colleagues, students, or employers. Do not forget this.

As in academic circles, a good reputation helps your wiki career. By logging in you can build a record of good edits, and it is easier to communicate and collaborate with others if you have a fixed, reputable identity. Finally, registering an account provides access to enhanced editing features, including a “watchlist” for monitoring articles you have edited previously.

## Rule 2: Learn the Five Pillars

There are some broad principles—known as the “five pillars”—all editors are expected to adhere to when contributing to Wikipedia. Perhaps most important for scientists is the appreciation that Wikipedia is not a publisher of original thought or research [9]. Accordingly, it is not an appropriate venue to promote your pet theory or share unpublished results. It is also not a soapbox on which to expound your personal theories or a battleground to debate controversial issues. In this respect, Wikipedia fundamentally differs from other types of new media, such as blogs, that encourage editorializing.

Contributing to Wikipedia is something to enjoy; a natural extension of your enthusiasm for science. But differences of opinion inevitably arise, particularly on pages provided for discussion on how to improve articles. Treat other editors as collaborators and maintain a respectful and civil manner, even in disagreement [10]. If you begin to find a particular interaction stressful, simply log off and come back another time. Unlike most scientific enterprises, Wikipedia has no deadlines.

## Rule 3: Be Bold, but Not Reckless

The survival and growth of any wiki requires participation. Wikipedia is unmatched in size, but its continuing success depends on the regular contributions of tens of thousands of volunteers. Therefore, Wikipedia urges all users to be bold: if you spot an error, correct it. If you can improve an article, please do so. It is important, however, to distinguish boldness from recklessness. Start off small. Begin by making minor modifications to existing articles before attempting a complete rewrite of *History of science*.

Many new editors feel intimidated about contributing to Wikipedia at first, fearing they may a mistake. Such reticence is understandable but unfounded. The worst that can happen is your first edits are deemed not to be an improvement and they get reverted. If this does occur, treat it as a positive learning experience and ask the reverting editor for advice.

## Rule 4: Know Your Audience

Wikipedia is not primarily aimed at experts; therefore, the level of technical detail in its articles must be balanced against the ability of non-experts to understand those details. When contributing scientific content, imagine you have been tasked with writing a comprehensive scientific review for a high school audience. It can be surprisingly challenging explaining complex ideas in an accessible, jargon-free manner. But it is worth the perseverance. You will reap the benefits when it comes to writing your next manuscript or teaching an undergraduate class.

## Rule 5: Do Not Infringe Copyright

With certain conditions, almost all of Wikipedia's content is free for anyone to reuse, adapt, and distribute. Consequently, it does not accept non-free material under copyright restriction. Some journals, including those from the Public Library of Science, publish material under an open-access license that is compatible with use in Wikipedia if properly attributed. Most do not. Therefore, although it may be tempting, avoid copying text or figures from your latest review article (or anyone else's) into Wikipedia. It will quickly be identified as a copyright violation and flagged for immediate deletion.

You can give Wikipedia permission to use material you own, but this process is non-reversible and can be time consuming. It is often better to rewrite the text in simpler language or redraw the figure to make it more accessible. This will also ensure it is more suitable for Wikipedia's non-expert readership (see Rule 4).

## Rule 6: Cite, Cite, Cite

To maintain the highest standards possible, Wikipedia has a strict inclusion policy that demands verifiability [11]. This is best established by attributing each statement in Wikipedia to a reliable, published source (but see Rules 7 and 8 on excessive self-citing). Most scientists are in the fortunate position of having access to a wide body of literature, and experience in using inline citations to support their writing. Since unverified content may be removed from Wikipedia at any time, provide supporting citations for every statement that might be challenged by another editor at some point in the future. Whenever possible, give preference to secondary sources (such as reviews or book chapters) that survey the relevant primary research over research articles themselves.

Wikipedia's accessibility makes each of its scientific articles an excellent entry point for laypeople seeking specialist information. By also providing direct hyperlinks to reliable, freely accessible online resources with your citations (biological databases or open-access journals, for example), other editors can quickly verify your content and readers have immediate access to authoritative sources that address the subject in greater detail.

## Rule 7: Avoid Shameless Self-Promotion

Many people are tempted to write or edit Wikipedia articles about themselves. Resist that urge. If you are sufficiently notable to merit inclusion in an encyclopedia, eventually someone else will write an article about you. Remember that unlike a personal Web page, your Wikipedia biography is not yours to control. A lovingly crafted hagiography extolling your many virtues can rapidly accumulate information you would rather not be publicized. You may already have a Wikipedia biography, but it contains factual inaccuracies that you wish to correct. How do you do this without breaking the rules? Wikipedia's guidelines encourage you to provide information about yourself on the associated discussion page, but please permit other editors to add it to the article itself.

Think twice, also, before writing about your mentors, colleagues, competitors, inventions, or projects. Doing so places you in a conflict of interest and inclines you towards unintentional bias [12]. If you have a personal or financial interest in the subject of *any* article you choose to edit, declare it on the associated discussion page and heed the advice of other editors who can offer a more objective perspective.

## Rule 8: Share Your Expertise, but Don't Argue from Authority

Writing about a subject about which you have academic expertise is not a conflict of interest [12]; indeed, this is where we can contribute to Wikipedia most effectively. Jimmy Wales, co-founder of Wikipedia, told *Nature* that experts have the ability to “write specifics in a nuanced way”, thereby significantly improving article quality [1]. When writing in your area of expertise, referencing material you have published in peer-reviewed journals is permitted if it is genuinely notable, but use common sense (and revisit Rule 7). For example, if you have an obscure, never-been-cited article in the *Journal of New Zealand Dairy Research* discussing the RNA content of cow milk, then referencing this in the introductory paragraph of the Wikipedia articles on “RNA”, “Milk”, “Cow”, and “Evolution of mammals” is not a good idea.

Occasionally you may interact with another editor who clearly does not share your expertise on the subject of an article. This can often prove frustrating for experts and is the basis of much academic angst on Wikipedia [1]. On such occasions, remember that you are assessed only on your contributions to Wikipedia, not who you are, your qualifications, or what you have achieved in your career. Your specialist knowledge should enable you to write in a neutral manner and produce reliable, independent sources to support each assertion you make. If you do not provide verification, your contributions will be rightly challenged irrespective of how many degrees you hold.

## Rule 9: Write Neutrally and with Due Weight

All articles in Wikipedia should be impartial in tone and content [13]. When writing, do state facts and facts about notable opinions, but do not offer *your* opinion as fact. Many newcomers to Wikipedia gravitate to articles on controversial issues about which people hold strong opposing viewpoints. Avoid these until familiar with Wikipedia's policies (see Rule 3), and instead focus on articles that are much easier to remain dispassionate about.

Many scientists who contribute to Wikipedia fail to appreciate that a neutral point of view is not the same as the mainstream scientific point of view. When writing about complex issues, try to cover all significant viewpoints and afford each with due weight, but not equal weight. For example, an article on a scientific controversy should describe both the scientific consensus and significant fringe theories, but not in the same depth or in a manner suggesting these viewpoints are equally held.

## Rule 10: Ask for Help

Wikipedia can be a confusing place for the inexperienced editor. Learning Wiki markup—the syntax that instructs the software how to render the page—may appear daunting at first, though the recent implementation of a new editing toolbar has made this easier, and usability development is ongoing. The intersecting guidelines and policies (and the annoying tendency of experienced editors to use an alphabet soup of acronyms to reference them) can also be tricky to comprehend. Thankfully, the Wikipedia community puts great stock in welcoming new editors. Guidance is available through a number of avenues, including help desks, a specific IRC channel, and an Adopt-a-User mentorship program. You can even summon help using a special template—{{helpme}}—and, as if by magic, a friendly Wikipedian will appear to offer one-on-one assistance.
